# Plasma metabolites in early adulthood are associated with midlife cognitive performance and brain tissue integrity in the CARDIA study

**DOI:** 10.1002/alz.71982

**Published:** 2026-11-09

**Authors:** Zsuzsanna Ament, Jelani Deajon‐Jackson, Amelia Demopoulos, Varun M. Bhave, Ana‐Lucia Garcia Guarniz, Ilya M. Nasrallah, Hemant K. Tiwari, M. Ryan Irvin, Venkatesh L. Murthy, Ravi Shah, Kristine Yaffe, R. Nick Bryan, W. Taylor Kimberly

**Affiliations:** ^1^ Harvard Medical School Boston Massachusetts USA; ^2^ Center for Genomic Medicine Massachusetts General Hospital Boston Massachusetts USA; ^3^ Department of Neurology Massachusetts General Brigham Boston Massachusetts USA; ^4^ Comprehensive Stroke Center Rhode Island Hospital Providence Rhode Island USA; ^5^ Department of Radiology Perelman School of Medicine, University of Pennsylvania Philadelphia Pennsylvania USA; ^6^ Department of Biostatistics, School of Public Health University of Alabama at Birmingham Birmingham Alabama USA; ^7^ Department of Epidemiology, School of Public Health University of Alabama at Birmingham Birmingham Alabama USA; ^8^ Division of Cardiovascular Medicine University of Michigan Ann Arbor Michigan USA; ^9^ Division of Cardiovascular Medicine Vanderbilt University Medical Center Nashville Tennessee USA; ^10^ UCSF Weill Institute for Neurosciences University of California San Francisco San Francisco California USA

**Keywords:** brain MRI, cognitive performance, global brain atrophy, metabolomics, white matter hyperintensity

## Abstract

**INTRODUCTION:**

Circulating plasma metabolites may serve as early indicators of midlife cognitive performance, but whether these associations are also reflected in midlife brain structure or white matter hyperintensity (WMH) burden remains unclear.

**METHODS:**

In the Coronary Artery Risk Development in Young Adults (CARDIA) study, we examined 575 plasma metabolites in early adulthood (Year 7; age [mean ± SD] 32 ± 3.6 years, *N =* 1,736) in relation to brain magnetic resonance imaging (MRI) and cognitive performance 23 years later (Year 30; age [mean ± SD] 55 ± 3.6 years, *N =* 646).

**RESULTS:**

Eleven metabolites were independently associated with cognition; five were also associated with total brain volume but none with WMH burden. In mediation analysis, total brain volume accounted for 8%–33% of metabolite–cognition associations.

**DISCUSSION:**

Plasma metabolites measured in early adulthood are associated with midlife cognitive performance and a subset is also associated with total brain volume, whereas no associations were observed with WMH burden, highlighting early‐adult metabolic signatures as markers of midlife brain structural health.

## Highlights

- Plasma metabolites in early adulthood were linked to cognition ≈20 years later
- Five cognition‐linked metabolites were also associated with total brain volume
- No metabolites were associated with white matter hyperintensity, suggesting non‐vascular pathways
- Total brain volume mediated the relationship between metabolites and cognitive performance

## 1 BACKGROUND

Identifying factors that indicate a future risk of cognitive decline is a priority for developing preventive strategies across the lifespan. Total brain volume loss captures cumulative tissue loss of both gray and white matter,^1^, ^2^, ^3^ secondary to aging, vascular, metabolic, and other processes, and is strongly associated with cognitive function and decline. White matter hyperintensity (WMH) burden is a radiological hallmark of microvascular dysfunction and small vessel disease,^4^, ^5^ which also correlates with cognitive decline.^6^ Although both total brain volume and WMH are each associated with cognitive performance, they are thought to reflect both shared and partially independent pathways, with the relative contribution of each still uncertain.^7^, ^8^, ^9^ We therefore focused on total brain volume and WMH burden as primary magnetic resonance imaging (MRI) phenotypes to examine complementary global structural and small‐vessel disease–related correlates of cognitive performance, and examined anatomically defined brain volumes to assess regional specificity.

Plasma metabolites capture the cumulative effects of diet, lifestyle, and metabolic processes,^10^ potentially providing early indicators of susceptibility to pathways relevant to brain health, including lipid metabolism, mitochondrial energy production, oxidative stress, and inflammation.^11^, ^12^, ^13^ Prior metabolite profiling studies suggest metabolic signatures measured before onset of pathology may relate to later cognitive outcomes.^13^, ^14^ However, it remains unclear whether early adult metabolic signatures are associated with later total brain volume, WMH burden, or cognitive performance through other pathways not captured through these measures. Here, we analyzed data from the Coronary Artery Risk Development in Young Adults (CARDIA)^15^ study to determine whether early adulthood metabolic profiles^16^ (age [mean ± SD] 32 ± 3.6 years) are associated with midlife structural brain changes^17^, ^18^ (age [mean ± SD] 55 ± 3.6 years), specifically total brain volume and WMH burden, and whether these changes mediate the relationship between early‐adult metabolites and cognitive performance in midlife (age [mean ± SD] 55 ± 3.6 years). A key strength of this design is that metabolites were measured in early adulthood, followed by midlife MRI and cognitive assessments, allowing metabolic profiles to be evaluated as early rather than contemporaneous correlates of midlife brain structure and cognitive performance.

## 2 METHODS

### 2.1 Study population

Details of participant recruitment, data collection, and retention across CARDIA study visits have been described previously.^15^ Briefly, CARDIA is a multicenter longitudinal study designed to measure risk factors for coronary artery disease in a cohort of Black and White women and men (*n =* 5,115), initially 18 to 30 years of age and healthy at enrollment (1985 to 1986). Participants were recruited from four U.S. cities (Birmingham, AL; Chicago, IL; Minneapolis, MN; and Oakland, CA), with each site approximately balanced by sex, age, race, and education.

### 2.2 Ethics

**RESEARCH IN CONTEXT**

1. **Systematic review**: Plasma metabolomics may capture early biological signatures relevant to later brain health. Prior studies have linked metabolites with risk of cognitive decline, but few have examined whether metabolic profiles in early adulthood are associated with later brain structure and cognitive performance, or whether brain structure helps explain these associations.
2. **Interpretation**: In Coronary Artery Risk Development in Young Adults (CARDIA) study, 11 plasma metabolites measured in early adulthood (≈32 years of age) were associated with cognitive performance approximately two decades later. Five were also associated with total brain volume, but none with white matter hyperintensity (WMH) burden. Mediation through total brain volume accounted for a modest proportion of these associations, suggesting that early metabolic profiles may be more closely linked to global brain tissue integrity than to WMH burden in midlife.
3. **Future directions**: Future studies should independently replicate these findings and include repeated metabolomic and neuroimaging measures, later‐life imaging when WMH is more prevalent, and amyloid and tau biomarkers to clarify Alzheimer's‐related pathways.

At each visit, participants provided written informed consent, and study protocols were reviewed by institutional review boards from each study site, the CARDIA Coordinating Center at the University of Alabama at Birmingham, and the University of California San Francisco. This study was conducted in accordance with the ethical standards of the 1964 Declaration of Helsinki and its later amendments. Further details of the study are available elsewhere.^15^, ^18^, ^19^ This manuscript follows the Strengthening the Reporting of Observational Studies in Epidemiology (STROBE) reporting guideline.^20^

### 2.3 Covariates

The CARDIA cohort was designed to recruit equal numbers of Black and White participants, men and women, and individuals across a range of ages and socioeconomic backgrounds, providing a racially and socioeconomically diverse sample that enhances the generalizability of findings to at‐risk populations for cardiometabolic and neurodegenerative disease. Analyzes were stratified or adjusted for race, sex, age, education, and study site to account for this heterogeneity. Age, sex, race, and education information collected via standardized CARDIA protocols based on participant self‐report. Race was categorized as Black or White. Education was recorded as years of completed schooling. The recruitment sites were included as a covariate to account for site‐level differences in recruitment and data collection. Cigarette smoking was defined as self‐reported smoking of at least five cigarettes per week, almost every week.^21^ Year 7 smoking status was categorized as never, former, or current smoking and was included in sensitivity analyzes as described below.

### 2.4 Cognitive function assessment

Study interviewers who underwent a centralized training administered a battery of three cognitive tests at the Year 30 examination (2015–2016). The Digit Symbol Substitution Test (DSST) was used to assess processing speed and executive function, with higher scores for digits correctly substituted indicating better function. The Stroop Test was used to assess executive function, with lower interference scores indicating better function (the inverse of this score was used, such that higher scores indicate better performance). The Rey Auditory Verbal Learning Test (RAVLT) assessed verbal memory, with higher scores after a 10‐min delay indicating better function.^18^ Test scores were converted to z‐scores using the entire Year 30 CARDIA cognition sample (*n =* 3147). We computed the composite cognitive z‐score as the mean of the three test‐specific z‐scores, with higher composite values indicating better overall cognitive performance across processing speed, executive function, and verbal memory.

### 2.5 MRI acquisition and processing

Brain MRI was conducted at the Year 30 examination of the CARDIA study (2015–016), following the standardized CARDIA protocol, including 1 mm isotropic T1‐weighted and T2 fluid‐attenuated inversion recovery (FLAIR) structural imaging. Acquisition parameters were harmonized across scanners and sites; full technical descriptions have been published previously.^19^, ^21^ All scans underwent centralized quality control, and data were processed with established automated processing pipelines to generate regional tissue volumes.^21^ Total brain volume and WMH burden were the primary MRI measures. Additional measures included cerebrospinal fluid (CSF), gray matter, white matter, corpus callosum, ventricular volume, and cerebellum volumes, and the deep white matter/gray matter ratio. These were examined in sensitivity analyses to assess how associations with cognitive performance varied across tissue compartments and anatomic structures. To assess regional specificity, we also examined frontal, parietal, temporal, occipital, and limbic gray matter volumes, and bilateral hippocampal volume. WMH volume was derived from FLAIR images, where these lesions appear as areas of increased signal intensity. All regional brain volumes were normalized to intracranial volume (ICV) by calculating volume to ICV ratios, and ratios were standardized to z‐scores for analysis. Because WMH exhibited a highly skewed distribution and included zero values, WMH ratios were log transformed after the addition of a small constant prior to standardization.

### 2.6 Metabolomics

Plasma metabolites at Year 7 in CARDIA (age [mean ± SD] 32 ± 3.5 years) were measured at the Broad Institute using established high‐resolution liquid chromatography tandem mass spectrometry (LC‐MS/MS) methods.^16^, ^22^, ^23^ Four complementary methods combined reversed‐phase (RP) chromatography and hydrophilic interaction liquid chromatography (HILIC) with positive or negative ionization metabolites were profiled using C8‐positive (lipids and nonpolar metabolites), C18‐negative (free fatty acids, lipid‐derived mediators, bile acids, and intermediate‐polarity metabolites), HILIC‐positive (amino acids, acylcarnitines, dipeptides, and other polar cations), and HILIC‐negative (sugars, organic acids, purines, pyrimidines, and other polar anions) approaches. Each participant sample was analyzed once per analytical method. Samples were analyzed in randomized order, with repeated measurements of pooled plasma controls interspersed throughout the analytical sequence to support normalization and assessment of analytical reproducibility. Detailed descriptions of sample extraction, chromatographic conditions, mass spectrometry parameters, and quality control procedures (including signal detection thresholds, deisotoping, and drift correction) have been published previously.^16^, ^22^, ^23^ The CARDIA Year 7 data release contained 612 curated metabolite, lipid, and internal‐standard features after upstream platform quality control. For the present analysis, we excluded 28 features with >10% missing observations, and 9 internal standards. The >10% missingness threshold restricted analyses to metabolites observed in at least 90% of participants and was intended to reduce the influence of poorly or intermittently measured signals. Missing values among retained metabolites were not imputed. Each model was fit on complete cases, resulting in small variation in sample size across models. The final analytic set comprised 575 metabolites: 207 detected by RP chromatography and 207 by HILIC in positive ionization mode, and 97 by RP and 64 by HILIC in negative ionization mode.

### 2.7 Statistics

Based on the study timeline (Figure 1A), four separate analytical groups were examined (Figure 1B). In the first group, we examined associations between total brain volume, WMH burden, and composite cognitive performance scores using linear regressions. In the second analytical group, we used linear regressions to identify metabolites associated with composite cognitive performance, applying platform‐specific Bonferroni‐corrected thresholds to account for the differing number of compounds detected per LC‐MS/MS method: *p <* 2.42×10^−4^ for RP positive‐mode and HILIC positive‐mode metabolites, *p <* 5.15×10^−4^ for RP negative‐mode, and *p <* 7.81×10^−4^ for HILIC negative‐mode metabolites. We applied Bonferroni rather than false discovery rate (FDR) correction to prioritize specificity given the exploratory‐to‐confirmatory aim; thresholds were computed within each platform/ionization mode to reflect the number of compounds tested per method. Eleven of 575 metabolites survived correction. In the third analytical group, linear regressions tested whether cognition‐associated metabolites were also associated with either total brain volume or WMH burden (*p <* 0.05). All models included age, race, sex, recruitment site, and years of education as covariates. Models did not adjust for cardiovascular risk factors (e.g., hypertension, diabetes), as these factors may lie on the causal pathway and/or interact with metabolic processes relevant to brain health.^24^, ^25^, ^26^ Adjusting for intermediates could induce overadjustment bias^27^, ^28^ and shift the interpretation toward controlled direct effects rather than total effects. Smoking was considered separately because it may influence both circulating metabolite profiles and later cognitive health. Smoking‐adjusted models were examined in sensitivity analyzes. The fourth analytical group tested mediation. For the mediation analyzes, we included all participants with complete metabolomics, cognitive testing, and MRI data (Figure 1B, sample 4). Metabolites associated with both cognition and brain volume measures were tested in mediation models (using the medeff Stata package^29^) adjusted for age, race, sex, recruitment site, and education; *p <* 0.05 was considered statistically significant. All analyses were conducted using StataSE v17. Figures were generated in RStudio (v2026.01.0).

**FIGURE 1:**
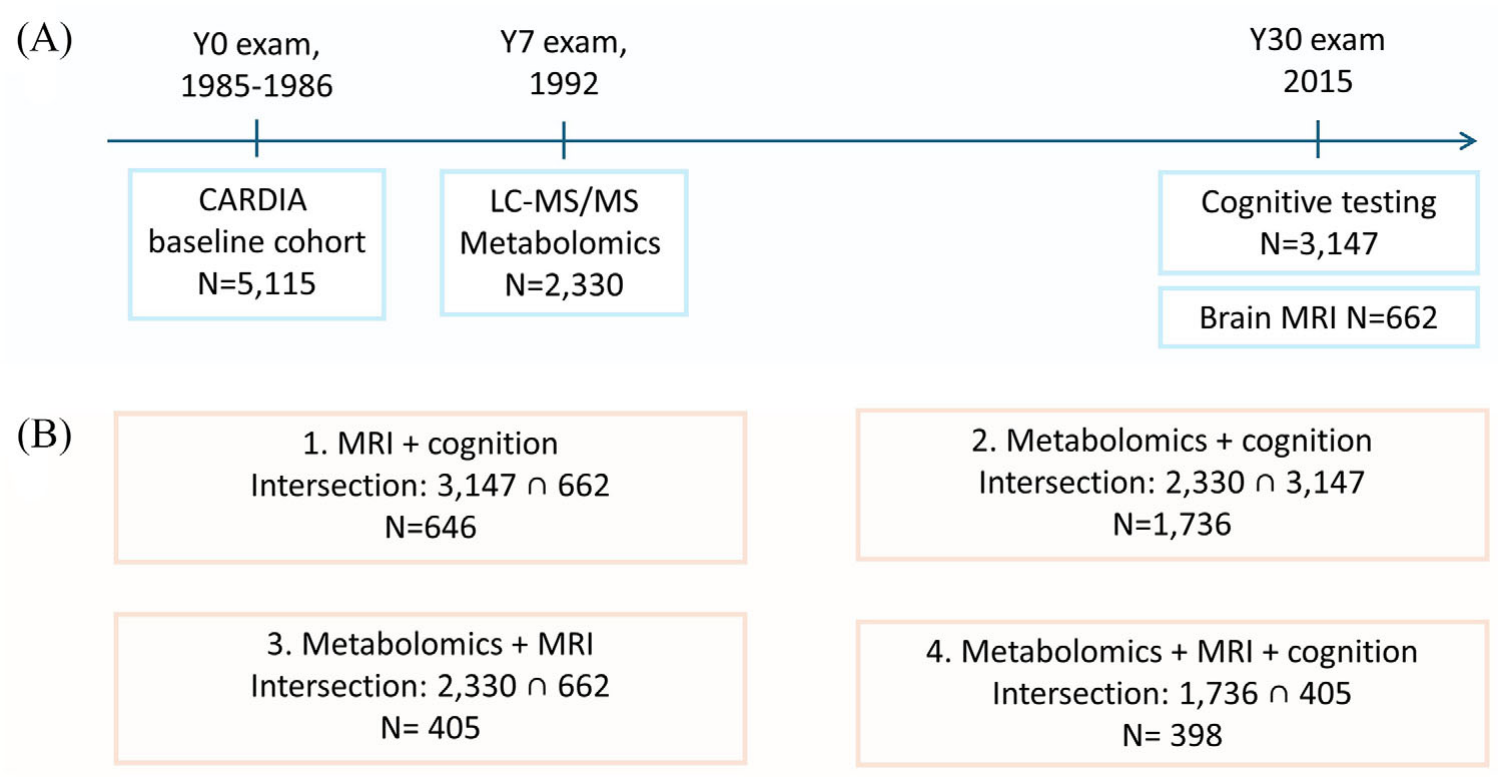
Overview of Coronary Artery Risk Development in Young Adults (CARDIA) study data used in the analyzes. (A) Shows the study timeline highlighting the exam years when plasma metabolomics were measured at Year 7, when comprehensive cognitive testing was administered at Year 30, and when brain magnetic resonance imaging (MRI) was completed at Year 30. (B) Lists the analytic sample sets used throughout the study: (1) participants with both cognitive testing and brain MRI at Year 30; (2) participants with metabolomics and cognitive testing; (3) participants with metabolomics and MRI; and (4) participants with metabolomics, cognition, and MRI included in the mediation analyses. Abbreviations: CARDIA = Coronary Artery Risk Development in Young Adults; LC‐MS/MS = liquid chromatography–tandem mass spectrometry; MRI = magnetic resonance imaging; Y0 = Year 0 (baseline); Y7 = Year 7; Y30 = Year 30.

### 2.8 Integrative metabolite analyses

Metabolites meeting the prespecified Bonferroni‐corrected thresholds for association with cognitive performance were carried forward for integrative analyses. To assess whether these metabolites represent shared biological pathways, we performed metabolite‐set over‐representation analysis using MetaboAnalyst 6.0,^30^ with all CARDIA‐measured metabolites as the reference background. Pairwise Spearman correlations were calculated among the candidate metabolites, with Benjamini–Hochberg FDR correction applied across all pairwise comparisons. Correlations with *q <* 0.05 were considered significant; for visualization, significant correlations with an absolute Spearman correlation coefficient ≥0.20 were displayed in the metabolite network.

### 2.9 Sensitivity analyses and effect–modification analyses

In sensitivity analyses, we assessed additional brain volume measures generated through the standardized CARDIA MRI analysis procedures.^19^, ^21^ Beyond the primary measures of total brain volume and WMH burden, we assessed CSF, gray matter, white matter, corpus callosum, cerebellum, ventricular, and deep white matter/gray matter ratio volumes, all normalized to ICV. We examined associations between these regional measures and individual cognitive domains (RAVLT, DSST, and inverse Stroop) using the same covariate‐adjusted linear regression models. To account for multiple comparisons across the nine regional measures within each cognitive domain, we applied a Bonferroni‐corrected threshold of *p <* 0.00556. In addition, we examined frontal, parietal, temporal, occipital, and limbic gray matter volumes, and hippocampal volume to assess whether associations involving cognition and brain structure showed regional anatomical specificity. Metabolites meeting the prespecified criteria for association with cognitive performance and the primary MRI measures were subsequently tested in relation to these regional volumes. Bonferroni correction was applied separately to the regional volume–cognition and metabolite–region analyses to account for multiple comparisons.

To evaluate sensitivity to smoking‐related exposure, primary metabolite–cognition models were repeated with additional adjustment for Year 7 smoking status, categorized as never, former, or current smoking. For metabolites eligible for mediation analysis under the prespecified criteria described, mediation models were also repeated with smoking included in both the mediator and outcome models. We also examined effect modification by apolipoprotein E (*APOE*) ε4 carrier status and race to assess whether metabolite associations varied across groups with potentially different susceptibility to cognitive and brain structural outcomes. *APOE* ε4 was examined because of its established role in cognitive decline and neurodegenerative disease, while race‐stratified analyses were used to evaluate the consistency of associations across the racially diverse CARDIA cohort. For metabolites carried forward from the primary cognition analysis, separate models included metabolite × *APOE* ε4 carrier status or metabolite × race interaction terms for cognitive performance and total brain volume. Subgroup‐specific estimates were obtained for *APOE* ε4 carriers and non‐carriers and for Black and White participants. Bonferroni correction was applied separately within each set of interaction tests to account for multiple comparisons.

## 3 RESULTS

### 3.1 Summary

In this study, we analyzed plasma metabolomics data (*N =* 2,330) measured at Year 7 (1992–1993; mean age 32 ± 3.6 years), and cognitive testing (*N =* 3,147) and MRI data (*N =* 662) collected at Year 30 (2015–2016). Analytic samples ranged in size from 1736 to 398 (Figure 1B; Table 1). Mean age at Year 30 was 55 years across all subgroups (SD 3.5), and composite cognitive z‐scores ranged from 0.06 to 0.12, indicating performance at or slightly above the overall Year 30 cohort average. The proportion of female participants was somewhat higher in the cognition‐only sample (52%) than in samples requiring metabolomics data (43%–47%), and the proportion of Black participants was modestly lower in metabolomics‐inclusive samples (36%–42%) compared to the cognition and MRI sample (40%). Full demographic characteristics are provided in Table 1.

**TABLE 1:** Characteristics of participants in each sample.

Variable	Sample 1 (Cognition + MRI) *N =* 646	Sample 2 (Metabolomics + cognition) *N =* 1736	Sample 3 (Metabolomics + MRI) *N =* 405	Sample 4 (Metabolomics + cognition + MRI) *N =* 398
Age at Y30, years (SD)	55.3 (3.5)	55.3 (3.5)	55.5 (3.5)	55.5 (3.5)
Female, *n* (%)	336 (52.0)	817 (47.1)	174 (43.0)	170 (42.7)
Black race, *n* (%)	258 (39.9)	736 (42.4)	148 (36.5)	145 (36.4)
Current smoker at Y30, *n* (%)	78 (12.2)	206 (12.0)	42 (10.5)	40 (10.2)
DSST, raw mean (SD)	68.8 (16.5)	68.5 (16.5)	69.0 (16.4)	69.0 (16.4)
RAVLT, mean (SD)	9.4 (2.0)	9.2 (1.9)	9.3 (2.0)	9.3 (2.0)
Stroop, mean (SD)	22.0 (11.2)	22.2 (11.2)	21.4 (10.8)	21.4 (10.8)
Composite cognitive z‐score, mean (SD)^*^	0.10 (0.80)	0.06 (0.78)	0.12 (0.78)	0.12 (0.78)
Education, years (SD)	14.1 (2.20)	14.3 (2.26)	14.3 (2.26)	14.3 (2.26)

Abbreviations: DSST, Digit Symbol Substitution Test; MRI, magnetic resonance imaging; RAVLT, Rey Auditory Verbal Learning Test; SD, standard deviation; Y30, Year 30.

* Composite cognitive score is the mean of z‐scored RAVLT, DSST, and inverted Stroop scores at Year 30.

### 3.2 Lower total brain volume and higher WMH burden are associated with worse cognition

We first examined whether total brain volume (reflecting cumulative brain tissue loss including vascular and neurodegenerative processes) and WMH burden (a marker of cerebral small vessel disease) were each associated with the composite cognitive performance at Year 30, consistent with prior CARDIA reports.^17^ Lower total brain volume was associated with worse cognitive performance (*β =* 0.12, 95% confidence interval [CI]: 0.06 to 0.17, *p =* 2.39×10^−5^), as was higher WMH burden (*β =* –0.12, 95% CI: –0.17 to –0.06, *p =* 1.43×10^−5^; Table 2). When both imaging markers were entered simultaneously into the same regression model, effect estimates for total brain volume (*β =* 0.13, 95% CI: 0.08 to 0.18, *p =* 1.56×10^−6^) and WMH burden (*β =* –0.13, 95% CI: –0.18 to –0.08, *p =* 1.13×10^−6^) were virtually unchanged, indicating that the two markers capture distinct variance in cognitive performance (Table 2).

**TABLE 2:** Associations of total brain volume and WMH burden with cognitive performance at Year 30.

MRI measure	β	95% CI low	95% CI high	*p*‐value
**Separate models**				
Total brain	0.12	0.06	0.17	2.39E‐05
WMH	−0.12	−0.17	−0.06	1.43E‐05
**Mutually adjusted model**				
Total brain	0.13	0.08	0.18	1.56E‐06
WMH	−0.13	−0.18	−0.08	1.13E‐06

*Note*: Linear regression of standardized cognition composite on standardized MRI measures (normalized to intracranial volume), adjusted for age, race, sex, recruitment site, and education (*N =* 646). Models examined each MRI measure separately and both measures together in relation to the cognitive composite score.

Abbreviations: CI, confidence interval; *β*, standardized regression coefficient; WMH, white matter hyperintensity.

Sensitivity analyses examining associations between cognitive performance and regional brain volumes (gray matter, white matter, corpus callosum, ventricular, cerebellar, and deep white matter/gray matter ratio) showed expected directional associations, but only total gray matter volume met the prespecified statistical threshold (*β =* 0.10, 95% CI: 0.04 to 0.15, *p =* 4.58×10^−4^). Domain‐specific associations varied across MRI measures (Table S1A). Among the six anatomically defined regional volumes, lower parietal (*β =* 0.10, 95% CI: 0.05 to 0.15, *p =* 2.07×10^−4^) and frontal volumes (*β =* 0.08, 95% CI: 0.03 to 0.14, *p =* 3.00×10^−3^) were associated with a worse composite cognitive performance score. The other four regional associations did not meet the Bonferroni‐corrected threshold. (Table S1B).

### 3.3 Early‐adulthood metabolites and midlife cognitive performance

We next assessed associations between plasma metabolites and future composite cognitive performance. Among 575 plasma metabolites, 11 were associated with composite cognitive scores (Figure 2, Table 3). Among these 11 metabolites, 7 showed positive associations, whereas 4 showed inverse associations. The strongest positive association was observed for glycerate (*β =* 0.09, 95% CI: 0.06 to 0.12, *p =* 1.72×10^−8^), a metabolite associated with higher fruit and vegetable intake,^31^ and involved in mitochondrial energy metabolism.^32^ The bioactive caffeine metabolite 3‐methylxanthine^33^ also showed a positive association (*β =* 0.07, *p =* 1.07×10^−4^). Metabolites linked to vitamin intake were positively associated, including C vitamin^34^ (erythronate/threonate, *β =* 0.07, *p =* 1.49×10^−5^) and B vitamin metabolism^35^ (4‐pyridoxate, *β =* 0.06, *p =* 6.89×10^−4^). Ribothymidine (5‐methyluridine), a modified nucleoside arising from RNA turnover and nucleoside metabolism,^36^ was also positively associated (*β =* 0.06, *p =* 1.45×10^−4^). The strongest inverse association was observed[Table alz71982-tbl-0003] for cotinine (*β =* –0.10, 95% CI: –0.14 to –0.07, *p =* 4.59×10^−9^), a nicotine metabolite and biomarker of tobacco exposure. The other metabolites with negative associations identified were lipids, specifically triacylglycerol (TAG): C56:5 (*β =* –0.06, *p* = 8.79×10^−5^), diacylglycerol (DAG): C38:5 (*β =* –0.06, *p =* 1.21×10^−4^), and sphingomyelin (SM) C24:1 (*β =* –0.06, *p =* 1.93×10^−4^). Overall, these metabolites point to diverse early‐adult metabolic signatures associated with midlife cognitive function. Because smoking may influence both circulating metabolite levels and later cognitive health, we examined the sensitivity of these associations to additional adjustment for Year 7 smoking status. Associations remained directionally consistent for all 11 metabolites. The greatest attenuation was observed for ribothymidine (59%) and cotinine (51%). Estimates for eight other metabolites were attenuated by ≈9% to 21%, whereas the C38:5 DAG estimate was essentially unchanged (Table S2).

**FIGURE 2:**
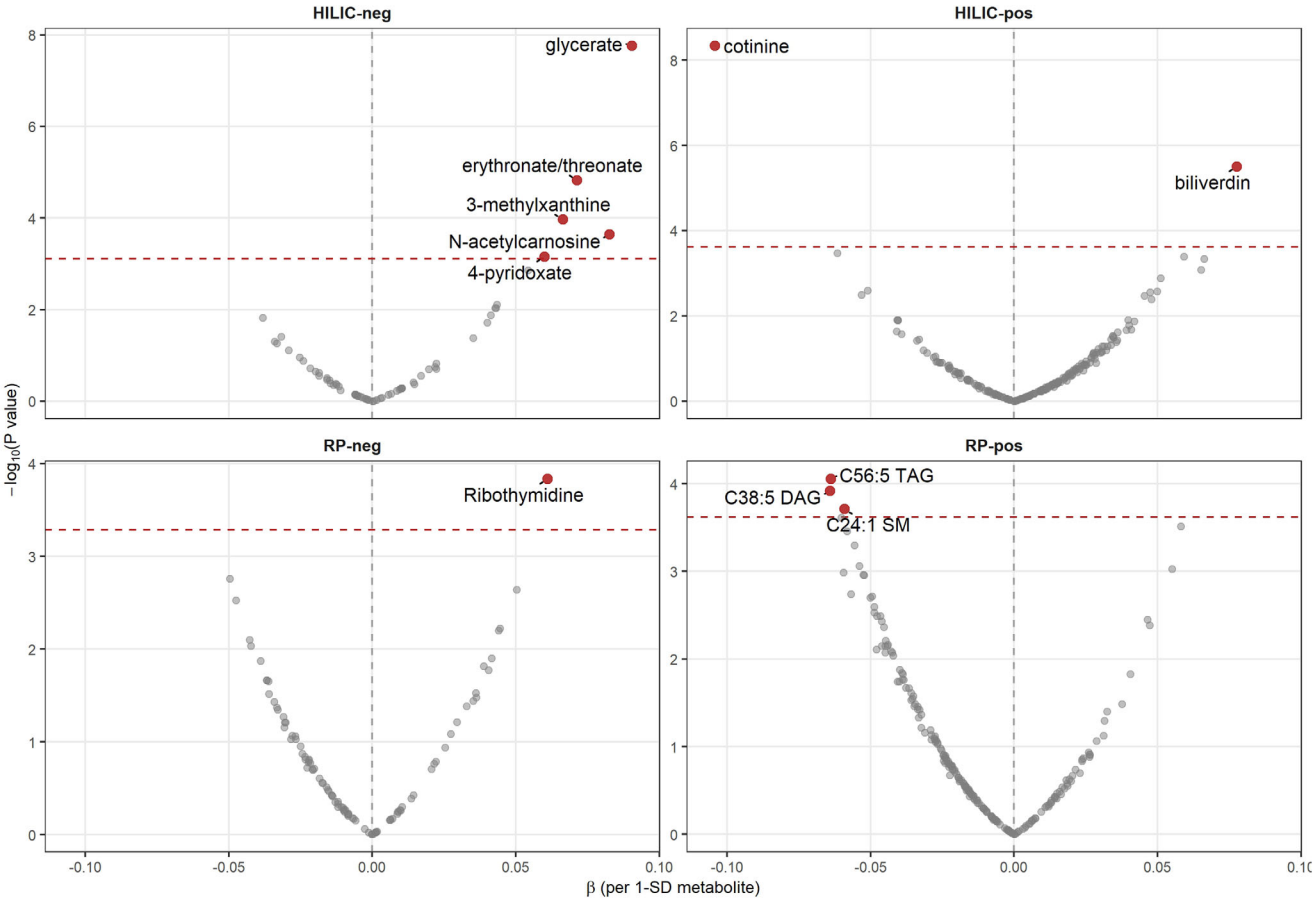
Volcano plot of plasma metabolites measured at Year 7 in association with composite measure of cognitive performance at Year 30. Each point represents a metabolite with effect estimates on the horizontal axis and negative log10 *p*‐values on the vertical axis. The cognitive composite was derived as the mean of standardized scores from three tests: the Digit Symbol Substitution Test (DSST), the Rey Auditory Verbal Learning Test (RAVLT), and the Stroop interference test (inverted, higher scores indicate better performance). Models are adjusted for age at examination, race, sex, recruitment site, and education. Metabolites meeting the Bonferroni significance threshold are highlighted. DAG, diacylglycerol; HILIC, hydrophilic interaction liquid chromatography; RP, reversed‐phase chromatography; SD, standard deviation; SM, sphingomyelin; TAG, triacylglycerol.

**TABLE 3 alz71982-tbl-0003:** Metabolites at Year 7 associated with Year 30 cognitive test scores.

Metabolite	β	95% CI low	95% CI high	*p*‐value
Glycerate (HILIC‐neg)	0.09	0.06	0.12	1.72E‐08
Cotinine (HILIC‐pos)	−0.10	−0.14	−0.07	4.59E‐09
Biliverdin (HILIC‐pos)	0.08	0.05	0.11	3.11E‐06
Erythronate/threonate (HILIC‐neg)	0.07	0.04	0.10	1.49E‐05
C56:5 TAG (RP‐pos)	−0.06	−0.10	−0.03	8.79E‐05
Ribothymidine (RP‐neg)	0.06	0.03	0.09	1.45E‐04
3‐Methylxanthine (HILIC‐neg)	0.07	0.03	0.10	1.07E‐04
C38:5 DAG (RP‐pos)	−0.06	−0.10	−0.03	1.21E‐04
C24:1 SM (RP‐pos)	−0.06	−0.09	−0.03	1.93E‐04
*N*‐acetylcarnosine (HILIC‐neg)	0.08	0.04	0.13	2.26E‐04
4‐pyridoxate (HILIC‐neg)	0.06	0.03	0.09	6.89E‐04

*Note*: Linear regression adjusted for age, race, sex, recruitment site, and education. Platform‐specific Bonferroni significance thresholds were applied: *p <* 2.42×10^−4^ for positive‐mode reversed‐phase chromatography (RP) and hydrophilic interaction liquid chromatography (HILIC) metabolites, *p <* 5.15×10^−4^ for negative‐mode RP, and *p <* 7.81×10^−4^ for negative‐mode HILIC metabolites.

### 3.4 Integrative analysis of cognition‐associated metabolites

Over‐representation analysis identified no metabolic pathways that remained significant after correction for multiple testing. Vitamin B6 metabolism was the highest‐ranked pathway at the nominal level but did not survive correction. Correlation analysis among the 11 cognition‐associated metabolites nevertheless revealed structured relationships. The strongest correlation was observed between C38:5 DAG and C56:5 TAG (*ρ =* 0.70), with C24:1 SM also connected to this lipid group. A second correlated group included glycerate, erythronate/threonate, and 4‐pyridoxate. Cotinine showed inverse correlations with several metabolites across these groups (Figure 3).

**FIGURE 3:**
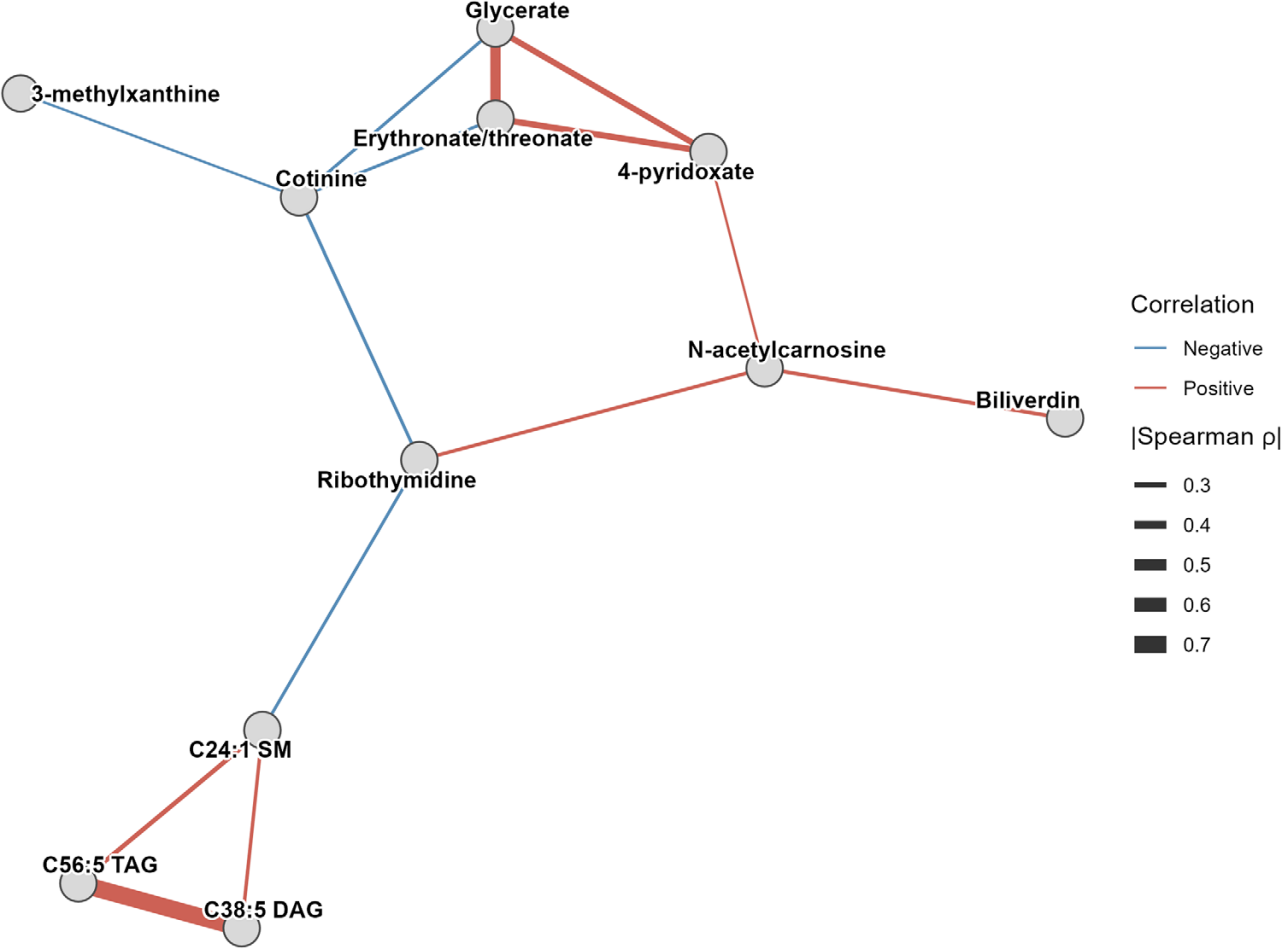
Correlation network of cognition‐associated metabolites. Spearman correlations were calculated among the 11 Year 7 metabolites associated with Year 30 cognitive performance. Edges represent correlations retained after correction for multiple comparisons. Red lines indicate positive correlations, and blue lines indicate inverse correlations; line width is proportional to the absolute Spearman correlation coefficient. False discover rate (FDR) *q <* 0.05 and |ρ|≥0.20. DAG, diacylglycerol; SM, sphingomyelin; TAG, triacylglycerol.

### 3.5 Effect modification by *APOE* ε4 carrier status and race

We found no evidence that *APOE* ε4 carrier status modified the associations of the 11 metabolites with cognitive performance after correction for multiple comparisons (all *p*’s for interaction ≥ 0.13). For total brain volume, glycerate and erythronate/threonate showed nominal interactions with *APOE* ε4 carrier status (*p* for interaction = 0.038 and 0.039, respectively). Similarly, nominal race interactions were observed for cognitive performance for cotinine, C38:5 DAG, 3‐methylxanthine, and biliverdin and for total brain volume for biliverdin, C38:5 DAG, erythronate/threonate, and glycerate, but none survived Bonferroni correction. Complete subgroup‐specific estimates are provided in Table S3.

### 3.6 Cognition‐associated metabolites relate to total brain volume but not WMH burden

To explore brain injury pathways that may be associated with cognition‐associated metabolites, the 11 candidate metabolites were next assessed in association with total brain volume and WMH burden. Five of the 11 metabolites showed associations with midlife total brain volume (nominal *p <* 0.05; Figure 4, Table S4). The strongest associations were observed for ribothymidine (*β =* 0.19, *p =* 8.07×10^−5^) and cotinine (*β =* –0.19, *p =* 9.61×10^−5^). Metabolites associated with total brain volume were directionally concordant with their cognitive associations: metabolites linked to poorer cognition were consistently linked to smaller total brain volumes. In contrast to total brain volume, we did not observe associations between metabolites and WMH volume (Table S4).

**FIGURE 4:**
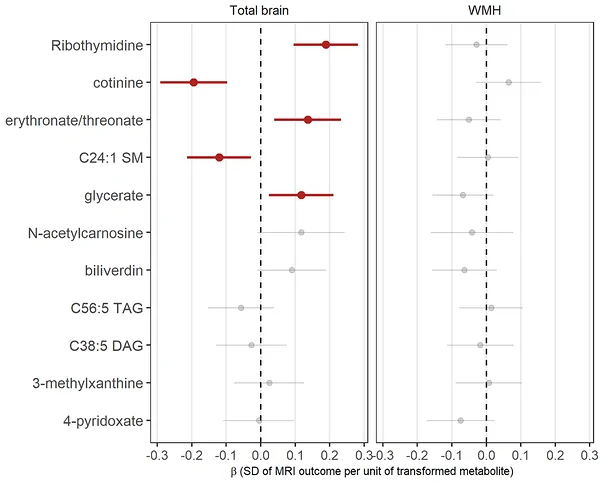
Forest plot of cognition associated metabolites and their associations with magnetic resonance imaging (MRI)–derived brain region volumes. Plot shows effect estimates and 95% confidence intervals for each cognition‐associated metabolite in relation to total brain volume and white matter hyperintensity burden. Estimates reflect regression models adjusted for age, race, sex, recruitment site, and education. DAG, diacylglycerol; MRI, magnetic resonance imaging; SD, standard deviation; SM, sphingomyelin; TAG, triacylglycerol; WMH = white matter hyperintensity.

Regional analyzes of the five metabolites associated with both cognition and total brain volume showed heterogeneous anatomic patterns rather than a common localized association. Of the 30 metabolite‐region comparisons, only ribothymidine's association with frontal gray matter volume survived Bonferroni correction (*β =* 0.16, *p =* 8.16×10^−4^). No consistent hippocampal or limbic signal was observed (Table S5).

### 3.7 Total brain volume partially mediates metabolite‐cognition associations

We next examined whether total brain volume mediates the association between the five metabolites associated with both total brain volume and cognitive performance. Total brain volume accounted for 8% to 33% of the metabolite‐cognition association (Figure 5, Table S6) For cotinine and C24:1 SM, indirect‐effect CIs excluded zero, whereas total‐effect CIs included zero. Their estimated percentages mediated should therefore be interpreted cautiously because the total effects used to calculate these proportions were imprecise estimates. In smoking‐adjusted mediation analyzes, indirect effects through total brain volume were modestly attenuated but remained significant for all five metabolites (Table S6). Overall, total brain volume accounted for a minority of the observed metabolite‐cognition associations, and this pattern was not materially altered by adjustment for smoking.

**FIGURE 5:**
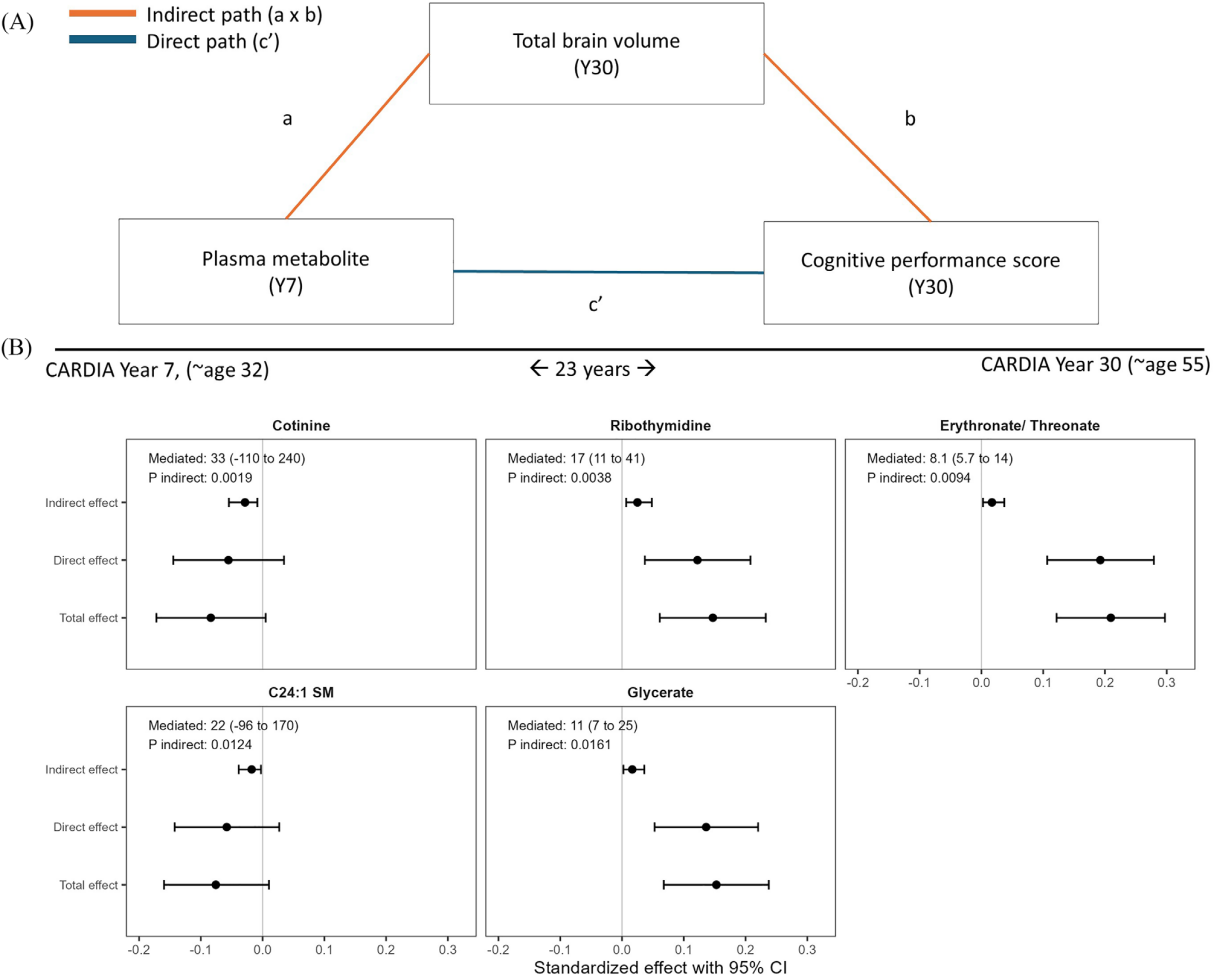
Brain volume partially mediates metabolite‐cognition associations. (A) Shows the conceptual path diagram illustrating the hypothesized mediation pathway from Year 7 plasma metabolites to Year 30 cognitive performance through total brain volume. (B) Shows mediation analysis results for metabolites with significant indirect effects. Points represent standardized regression coefficients and horizontal lines represent 95% confidence intervals, based on 10,000 quasi‐Bayesian simulations. The indirect effect (top row) reflects the pathway from metabolite to cognition operating through total brain volume (path a × b); the direct effect (middle row) reflects the remaining association independent of brain volume; and the total effect (bottom row) is their sum. “Mediated” denotes the proportion of the total effect explained by the indirect pathway. These proportions should be interpreted cautiously for cotinine and C24:1 sphingomyelin (SM), whose total‐effect confidence intervals include zero. The *p*‐value shown refers to the test of the indirect effect. The mediator was the standardized total brain volume/intracranial volume ratio. All models adjusted for age, race, sex, recruitment site, and education. Mediator and outcome regressions included 405 and 398 participants, respectively, except for cotinine (404 and 397). Full results are provided in Table S6. CARDIA, Coronary Artery Risk Development in Young Adults; CI, confidence interval; SM, sphingomyelin; Y7, Year 7; Y30, Year 30.

## 4 DISCUSSION

In this retrospective study, we found that metabolic profiles measured in early adulthood are associated with midlife cognitive performance, and these associations are stronger with global brain atrophy than with microvascular injury. Both WMH burden and total brain volume were independently associated with cognitive performance at midlife, suggesting that both microvascular injury and global brain tissue loss contribute to midlife cognitive function. Several metabolites were associated with cognitive performance and total brain volume, but no associations were detected with WMH burden. Total brain volume accounted for 8%–33% of these associations, indicating that the link between early‐adult metabolites and midlife cognition is not entirely captured by macrostructural brain volume.

### 4.1 Metabolite associations with midlife brain structure and WMH burden

The differences between metabolite associations with total brain volume but not WMH burden suggest that early‐adult metabolic profiles may be less strongly linked to focal white matter lesion accumulation characteristic of small vessel disease. Five of the 11 cognition‐associated metabolites were also directly associated with total brain volume. However, total brain volume is not a specific marker of any single pathological process, instead providing an aggregate measure influenced by neuronal, vascular, developmental, and other factors. The regional analyses provided limited evidence for anatomic specificity in the metabolite–brain volume associations. Some correlated metabolites nevertheless showed similar regional patterns. In particular, glycerate and erythronate/threonate varied together across participants and showed similar associations with parietal and occipital gray matter volumes. However, these patterns remain exploratory and do not establish a shared regional signature. Together, these findings suggest broadly distributed differences in brain structure, with limited evidence for a consistent regional pattern. Total brain volume partially accounted for the metabolite–cognition associations, suggesting that global brain structure represents one of several pathways linking early adult metabolic profiles with later cognitive performance. The age of the CARDIA cohort is also relevant to interpreting these findings. At mean age 55 at the time of MRI and cognitive assessments, Alzheimer's disease (AD) pathologic change is expected to be rare and, if present, minimal in burden. Because total brain volume is a nonspecific phenotype integrating diverse pathologies, we cannot attribute these differences to a single process, and because metabolites showed no association with WMH, overt small vessel disease is also unlikely to be the primary driver. WMH is a common and sensitive imaging marker of cerebral small vessel disease, but it does not capture the full spectrum of vascular brain injury.^37^ The absence of metabolite–WMH associations may reflect the timing of assessment. Metabolites may not predict discrete vascular lesions that develop decades later if the WMH accumulation falls outside our measurement window, even if they are associated with other aspects of midlife brain structure. Recent evidence demonstrates that white matter changes follow non‐linear trajectories across the lifespan, with WMH burden showing particularly rapid accumulation after age 60.^38^ Our WMH assessment at a mean age of 53 (range 48–60) may precede the period of steepest accumulation for many participants.^39^ Consistent with this, observed WMH was low in our sample (median 0.56 mL, interquartile range [IQR] 0.24–1.23 mL). Metabolites measured at mean age 32 may predict processes that manifest more clearly during later WMH acceleration.

### 4.2 Integrative interpretation of the metabolic signature

The metabolic signature appears to reflect several distinct aspects of metabolic and behavioral health rather than a single biochemical pathway, as the identified metabolites did not converge on a common metabolic pathway. However, the identified metabolites represent biologically interpretable domains. The correlated lipid species, including C56:5 TAG, C38:5 DAG, and C24:1 SM, were consistently associated with poorer cognitive performance, suggesting that variation in lipid handling and membrane lipid metabolism may be one component of the observed signature. C24:1 SM was also associated with lower total brain volume, providing concordant structural and cognitive evidence for this lipid‐related pattern. In contrast, glycerate, erythronate/threonate, and 4‐pyridoxate clustered together and were positively associated with cognitive performance. These metabolites may reflect nutritional and intermediary metabolic profiles, with glycerate previously associated with greater fruit and vegetable intake, erythronate/threonate linked to vitamin C metabolism, and 4‐pyridoxate reflecting vitamin B6 metabolism. Glycerate and erythronate/threonate were also associated with larger total brain volume, providing structural associations concordant with their cognitive associations for these metabolites. Cotinine is a highly specific marker of tobacco and nicotine exposure. Higher levels were associated with both poorer cognition and lower total brain volume. Adjustment for smoking status attenuated the cognitive association by 51%, indicating that cotinine captures aspects of exposure not reflected in self‐reported smoking status. Higher ribothymidine, a modified nucleoside related to RNA turnover, was likewise associated with both better cognition and larger total brain volume. Its association with cognition was substantially attenuated after adjustment for smoking status, suggesting that it may partly reflect smoking‐related behavioral or metabolic differences. However, the indirect association through total brain volume persisted in the MRI sample after adjustment, suggesting that reported smoking status did not fully explain this association. Together, these findings give the metabolite signature a clear biological context: tobacco exposure, nutrition, and lipid metabolism in early adulthood. The associations with later cognition and brain volume suggest that these aspects of behavioral and metabolic health are relevant well before midlife. The signature therefore identifies early metabolic correlates of later brain health, whereas the mechanisms underlying these associations remain to be established.

### 4.3 Total brain volume as a potential mediator

Metabolites were measured ≈23 years before the Year 30 MRI and cognitive assessments, establishing that the metabolic measures preceded both outcomes. However, because brain MRI and cognition were assessed at the same visit, the temporal ordering between brain structure and cognitive performance cannot be established. Because total brain volume partially mediated each association, these relationships likely include other pathways in addition to volumetric MRI, plausibly microstructural white matter changes, synaptic and neurochemical effects, and neurovascular mechanisms, as well as cognitive reserve. Of interest, some of the metabolites identified are implicated in health‐related habits (such as diet and smoking), suggesting that these long‐term exposures have measurable impacts on brain health as early as midlife.

### 4.4 Strengths and limitations

This study has several strengths. It is a well‐characterized longitudinal cohort with information across understudied points in the lifespan, including early and midlife adulthood, and uses a comprehensive range of untargeted metabolomics methods that captured a broad range of metabolic pathways across 575 metabolites. However, there are also limitations. Metabolomics detection methods remain incomplete, due to inherent limitations in chromatographic separation and mass spectrometry (MS) detection. Metabolites were measured at a single timepoint at Year 7 of the CARDIA study and does not capture longer term fluctuations in metabolic exposures. By design, brain MRI and cognitive testing were assessed at Year 30, reflecting a single cross‐sectional timepoint. Although the absence of metabolite WMH associations may reflect genuine biological independence, it could also relate to the timing of measurement if WMH had not yet accumulated, or to limited sensitivity to detect subtle early microvascular changes. In addition, the long interval between metabolite measurement and brain imaging may have reduced the ability to detect associations. Future studies with repeated MRI assessments across the sixth and seventh decades, when WMH burden accelerates, may better characterize metabolite microvascular relationships. Repeated metabolic profiling, assessed closer in time to imaging, would further strengthen these inferences. Because brain MRI was cross‐sectional, lower midlife total brain volume may partly reflect differences in peak (developmentally attained) brain volume rather than tissue loss over adulthood; our design cannot distinguish these, and the associations should be read as differences in attained structure rather than measured atrophy. Findings derive from a single cohort and have not yet been replicated in an independent population. However, the unique combination of metabolomic profiling in early adulthood and cognitive and neuroimaging assessment in midlife limits the availability of comparable replication cohorts. Finally, analyses of the effects of *APOE* ε4 carrier status and race may have been underpowered, and larger studies will be needed to determine whether true heterogeneity exists.

## 5 CONCLUSIONS

In this prospective cohort of U.S. adults followed for more than two decades, early adulthood plasma metabolites were associated with midlife cognitive performance, and for a subset of metabolites. with total brain volume. Total brain volume partially accounted for the associations, whereas no associations were observed with WMH burden. These findings highlight that early adult metabolic signatures, particularly those reflecting environmental, behavioral, and dietary exposures such as tobacco use and fruit and vegetable intake are detectable more than two decades earlier. Together, these observations suggest that modifiable lifestyle factors may influence cognitive performance decades later, partly through their effects on brain tissue integrity.

## CONSENT STATEMENT

All participants provided written informed consent at each study examination. The study protocol was approved by the institutional review boards at each participating center.

## CONFLICT OF INTEREST STATEMENT

Z. Ament, J. Deajon‐Jackson, A. Demopoulos, V.M. Bhave, A. Garcia Guarniz, I.M. Nasrallah, H.K. Tiwari, and M.R. Irvin report no relevant disclosures. K. Yaffe serves on Data Safety Monitoring Boards for Eli Lilly and several National Institute on Aging–sponsored studies, is a board member of Alector, and is a member of the Beeson Scientific Advisory Board and the Global Council on Brain Health. V.L. Murthy owns stock or stock options in Abbott Laboratories, AbbVie, Advanced Micro Devices, Amgen, Baxter International, Boston Scientific, Bristol‐Myers Squibb, Cardinal Health, Cigna, Eli Lilly, GE Healthcare, Intel, Ionetix, Johnson & Johnson, Medtronic, Merck, nVidia, Oracle, Pfizer, Viatris, and Zimmer Biomet. He has received research grants and consulting fees from Siemens Medical Imaging, and consulting fees from Jubilant Draximage and Credence Management Solutions. He has served on a medical advisory board for Ionetix. R. Shah has received support from Amgen (scientific advisory board) and is a co‐inventor on patents and pending patent applications related to proteomics, metabolomics biomarkers, and RNA‐based diagnostics. W.T. Kimberly has received consulting fees for Scientific Advisory Board services (Takeda, EMD Serono, Corxel, Astrocyte, and BMS‐J&J Alliance) and for Data and Safety Monitoring Board services (Stryker). Author disclosures are available in the Supporting Information.

## Supporting information




**Supporting Information**: alz71982‐sup‐0001‐Table S1‐S6.docx

**Supporting Information**: alz71982‐sup‐0002‐ICMJE.pdf
