# Supplementary material for: Plasma metabolites in early adulthood are associated with midlife cognitive performance and brain tissue integrity in the CARDIA study
Source: Alzheimers Dement. 2026 Nov 9;22(11):e71982. doi: 10.1002/alz.71982 (PMC13746177; doi:10.1002/alz.71982)
Supplement: Supplementary file 2 [file ALZ-22-e71982-s002.docx]

**Table S1.** Sensitivity analyses of brain MRI measures and cognitive performance. Linear regression models examined associations between standardized brain MRI measures and standardized cognitive outcomes at Year 30 (N=646), adjusted for age, race, sex, recruitment site, and education**. Panel A** includes global and tissue-compartment MRI and their associations with the composite cognitive score and domain specific measures (RAVLT, DSST, and inverse Stroop). **Panel B** includes anatomically defined regional grey matter volumes and hippocampal volume with their associations with the composite cognitive score. All MRI volumes were normalized to intracranial volume. Bonferroni corrected thresholds were applied separately within each panel: **in Panel A** P < 0.00556 **for the nine MRI measures assessed and in Panel B P < 0.00833 for the six defined regional volumes assessed.**

| **Panel A. Global and tissue-compartment measures** | | | | | |
| --- | --- | --- | --- | --- | --- |
|  | **Brain region volume** | **β** | **95% CI low** | **95% CI high** | **P value** |
| **Composite (RAVLT, DSST, inv-Stroop)** | **Total brain** | **0.116** | **0.062** | **0.169** | **2.39E-05** |
|  | **WMH** | **-0.117** | **-0.170** | **-0.065** | **1.43E-05** |
|  | **CSF** | **-0.115** | **-0.169** | **-0.062** | **2.54E-05** |
|  | **Gray matter** | **0.097** | **0.043** | **0.151** | **4.58E-04** |
|  | White matter | 0.060 | 0.009 | 0.110 | 2.03E-02 |
|  | Corpus callosum | 0.045 | -0.005 | 0.095 | 7.72E-02 |
|  | Ventricular | -0.050 | -0.101 | 0.002 | 5.85E-02 |
|  | Cerebellar | 0.042 | -0.011 | 0.095 | 1.16E-01 |
|  | Deep WM to GM ratio | 0.061 | 0.004 | 0.118 | 3.57E-02 |
|  |  |  |  |  |  |
| **RAVLT** | Total brain | 0.097 | 0.024 | 0.171 | 9.62E-03 |
|  | **WMH** | **-0.117** | **-0.190** | **-0.044** | **1.78E-03** |
|  | CSF | -0.098 | -0.172 | -0.024 | 9.36E-03 |
|  | Gray matter | 0.099 | 0.025 | 0.173 | 8.56E-03 |
|  | White matter | 0.026 | -0.043 | 0.095 | 4.65E-01 |
|  | Corpus callosum | 0.057 | -0.011 | 0.126 | 9.98E-02 |
|  | Ventricular | -0.014 | -0.085 | 0.056 | 6.92E-01 |
|  | Cerebellar | 0.044 | -0.029 | 0.116 | 2.36E-01 |
|  | Deep WM to GM ratio | 0.104 | 0.027 | 0.182 | 8.41E-03 |
|  |  |  |  |  |  |
| **DSST** | **Total brain** | **0.121** | **0.052** | **0.190** | **5.79E-04** |
|  | WMH | **-0.078** | **-0.146** | **-0.011** | **2.31E-02** |
|  | **CSF** | **-0.121** | **-0.189** | **-0.052** | **6.13E-04** |
|  | Gray matter | 0.083 | 0.014 | 0.153 | 1.90E-02 |
|  | White matter | 0.087 | 0.022 | 0.152 | 8.36E-03 |
|  | Corpus callosum | 0.064 | 0.000 | 0.128 | 4.97E-02 |
|  | Ventricular | -0.053 | -0.119 | 0.013 | 1.15E-01 |
|  | Cerebellar | 0.058 | -0.010 | 0.126 | 9.18E-02 |
|  | Deep WM to GM ratio | 0.035 | -0.038 | 0.108 | 3.43E-01 |
|  |  |  |  |  |  |
| **Stroop (inverse)** | **Total brain** | **0.134** | **0.059** | **0.208** | **4.45E-04** |
|  | **WMH** | **-0.139** | **-0.213** | **-0.065** | **2.46E-04** |
|  | **CSF** | **-0.133** | **-0.208** | **-0.058** | **4.98E-04** |
|  | Gray matter | 0.108 | 0.033 | 0.184 | 4.97E-03 |
|  | White matter | 0.076 | 0.006 | 0.147 | 3.44E-02 |
|  | Corpus callosum | 0.018 | -0.052 | 0.088 | 6.10E-01 |
|  | Ventricular | -0.084 | -0.156 | -0.012 | 2.20E-02 |
|  | Cerebellar | 0.019 | -0.054 | 0.093 | 6.07E-01 |
|  | Deep WM to GM ratio | 0.049 | -0.031 | 0.129 | 2.29E-01 |
|  | | | | | |
| **Panel B. Anatomically defined regional gray matter volumes** | | | | | |
|  | **Brain region volume** | **β** | **95% CI low** | **95% CI high** | **P value** |
| **Composite** | **Parietal** | **0.101** | **0.048** | **0.154** | **2.07E-04** |
|  | **Frontal** | **0.083** | **0.029** | **0.136** | **3.00E-03** |
|  | Temporal | 0.061 | 0.010 | 0.111 | 1.80E-02 |
|  | Occipital | 0.051 | -0.001 | 0.103 | 5.30E-02 |
|  | Limbic | 0.033 | -0.019 | 0.084 | 2.15E-01 |
|  | Hippocampal | -0.032 | -0.085 | 0.021 | 2.36E-01 |

**Table S2**. Sensitivity analyses after adjustment for Year 7 smoking status. Associations between the 11 cognition-associated metabolites and cognitive performance were assessed before and after adjustment for smoking status. Attenuation represents the percentage reduction in the absolute effect estimate after adjustment; positive values indicate weaker associations, and negative values indicate stronger associations. Primary models were adjusted for age, race, sex, recruitment site, and education. Smoking-adjusted models additionally included Year 7 smoking status, categorized as never, former, or current.

| **Metabolite** | **Primary  N** | **Primary  β** | **95% CI** | | **Smoking-adjusted N** | **Smoking-adjusted β** | **95% CI** | | **Attenuation %β** |
| --- | --- | --- | --- | --- | --- | --- | --- | --- | --- |
| 3-Methylxanthine | 1600 | 0.066 | 0.03 | 0.10 | 1597 | 0.054 | 0.02 | 0.09 | 18.2 |
| 4-Pyridoxate | 1731 | 0.060 | 0.03 | 0.09 | 1728 | 0.050 | 0.02 | 0.08 | 16.7 |
| Biliverdin | 1736 | 0.078 | 0.05 | 0.11 | 1733 | 0.062 | 0.03 | 0.10 | 20.5 |
| C24:1 SM | 1736 | -0.059 | -0.09 | -0.03 | 1733 | -0.054 | -0.09 | -0.02 | 8.5 |
| C38:5 DAG | 1736 | -0.064 | -0.10 | -0.03 | 1733 | -0.065 | -0.10 | -0.03 | -1.6 |
| C56:5 TAG | 1736 | -0.064 | -0.10 | -0.03 | 1733 | -0.055 | -0.09 | -0.02 | 14.1 |
| Cotinine | 1733 | -0.104 | -0.14 | -0.07 | 1730 | -0.051 | -0.10 | -0.01 | 51.0 |
| Erythronate/ Threonate | 1736 | 0.071 | 0.04 | 0.10 | 1733 | 0.059 | 0.03 | 0.09 | 16.9 |
| Glycerate | 1736 | 0.090 | 0.06 | 0.12 | 1733 | 0.080 | 0.05 | 0.11 | 11.1 |
| N-Acetylcarnosine | 1731 | 0.083 | 0.04 | 0.13 | 1728 | 0.066 | 0.02 | 0.11 | 20.5 |
| Ribothymidine | 1735 | 0.061 | 0.03 | 0.09 | 1732 | 0.025 | -0.01 | 0.06 | 59.0 |

**Table S3**. Associations of cognition-related metabolites with total brain volume and cognitive performance by APOE ε4 carrier status and race**.** Linear regression models examined the 11 Year 7 cognition-associated metabolites in relation to Year 30 total brain volume and composite cognitive performance. **Panels A and B** present associations by APOE ε4 carrier status for total brain volume and cognition, respectively; **Panels C and D** present corresponding associations by race. Models included age, sex, recruitment site, education, race, and the relevant metabolite × APOE ε4 or metabolite × race interaction term. Interaction coefficients represent differences in metabolite associations between ε4 carriers and non-carriers or between White and Black participants. Bonferroni correction was applied across 11 interaction tests within each panel (P < 0.00455). No interaction met this threshold.

| **Panel A. Associations with total brain volume by APOE ε4 carrier status** | | |  |  |  |  |
| --- | --- | --- | --- | --- | --- | --- |
| **Metabolite** | **ε4 non-carrier β**  **(95% CI)** | **P** | **ε4 carrier β  (95% CI)** | **P** | **Interaction β**  **(95% CI)** | **P interaction** |
| Y7 cotinine  (HILIC-pos) | -0.204 (-0.319, -0.089) | 0.0005 | -0.185 (-0.348, -0.022) | 0.0260 | 0.019 (-0.171, 0.21) | 0.8400 |
| Y7 biliverdin  (HILIC-pos) | 0.115 (-0.008, 0.238) | 0.0660 | 0.092  (-0.077, 0.26) | 0.2900 | -0.024 (-0.226, 0.179) | 0.8200 |
| Y7 C24:1 SM  (C8-pos) | -0.122 (-0.23, -0.013) | 0.0280 | -0.131  (-0.306, 0.044) | 0.1400 | -0.009  (-0.212, 0.193) | 0.9300 |
| Y7 C38:5 DAG  (C8-pos) | -0.021  (-0.139, 0.098) | 0.7300 | -0.036  (-0.23, 0.159) | 0.7200 | -0.015  (-0.239, 0.209) | 0.8900 |
| Y7 C56:5 TAG  (C8-pos) | -0.044 (-0.158, 0.07) | 0.4500 | -0.081 (-0.262, 0.1) | 0.3800 | -0.037  (-0.249, 0.176) | 0.7300 |
| Y7 Ribothymidine  (C18-neg) | 0.201 (0.086, 0.316) | 0.0006 | 0.183  (0.02, 0.345) | 0.0280 | -0.019  (-0.214, 0.177) | 0.8500 |
| Y7 3-methylxanthine (HILIC-neg) | 0.025 (-0.089, 0.139) | 0.6700 | 0.067  (-0.135, 0.269) | 0.5200 | 0.042  (-0.183, 0.267) | 0.7100 |
| Y7 4-pyridoxate  (HILIC-neg) | 0.029 (-0.093, 0.152) | 0.6400 | -0.08  (-0.247, 0.087) | 0.3500 | -0.109  (-0.304, 0.086) | 0.2700 |
| erythronate/threonate (HILIC-neg) | 0.079  (-0.031, 0.189) | 0.1600 | 0.307  (0.115, 0.5) | 0.0018 | 0.228 (0.012, 0.445) | 0.0390 |
| Y7 glycerate  (HILIC-neg) | 0.053  (-0.057, 0.164) | 0.3400 | 0.266  (0.093, 0.44) | 0.0027 | 0.213  (0.012, 0.414) | 0.0380 |
| Y7 N-acetylcarnosine (HILIC-neg) | 0.141  (0.003, 0.279) | 0.0460 | 0.063 (-0.131, 0.258) | 0.5200 | -0.078 (-0.28, 0.124) | 0.4500 |
|  |  |  |  |  |  |  |
| **Panel B. Associations with cognitive performance by APOE ε4 carrier status** | | |  |  |  |  |
| **Metabolite** | **ε4 non-carrier β  (95% CI)** | **P** | **ε4 carrier β (95% CI)** | **P** | **Interaction β**  **(95% CI)** | **P interaction** |
| Y7 cotinine  (HILIC-pos) | -0.111 (-0.151, -0.071) | 6.1E-08 | -0.096  (-0.158, -0.033) | 0.0028 | 0.015 (-0.055, 0.086) | 0.6700 |
| Y7 biliverdin  (HILIC-pos) | 0.074 (0.035, 0.113) | 0.0002 | 0.089 (0.034, 0.144) | 0.0016 | 0.015 (-0.05, 0.08) | 0.6500 |
| Y7 C24:1 SM  (C8-pos) | -0.064 (-0.101, -0.026) | 0.0009 | -0.044  (-0.1, 0.012) | 0.1300 | 0.02 (-0.047, 0.086) | 0.5600 |
| Y7 C38:5 DAG  (C8-pos) | -0.073  (-0.111, -0.035) | 0.0002 | -0.024  (-0.087, 0.039) | 0.4600 | 0.05 (-0.023, 0.122) | 0.1800 |
| Y7 C56:5 TAG  (C8-pos) | -0.073  (-0.109, -0.036) | 0.0001 | -0.03  (-0.092, 0.032) | 0.3400 | 0.042  (-0.029, 0.113) | 0.2400 |
| Y7 Ribothymidine  (C18-neg) | 0.078  (0.04, 0.115) | 4.8E-05 | 0.027  (-0.031, 0.084) | 0.3600 | -0.051  (-0.119, 0.016) | 0.1400 |
| Y7 3-methylxanthine (HILIC-neg) | 0.063  (0.025, 0.102) | 0.0013 | 0.073  (0.008, 0.137) | 0.0270 | 0.01  (-0.064, 0.083) | 0.8000 |
| Y7 4-pyridoxate  (HILIC-neg) | 0.061 (0.021, 0.101) | 0.0028 | 0.066 (0.006, 0.126) | 0.0300 | 0.005 (-0.063, 0.073) | 0.8800 |
| erythronate/threonate (HILIC-neg) | 0.087  (0.049, 0.126) | 7.0E-06 | 0.035  (-0.023, 0.092) | 0.2400 | -0.053  (-0.12, 0.015) | 0.1300 |
| Y7 glycerate  (HILIC-neg) | 0.104  (0.067, 0.141) | 4.5E-08 | 0.068  (0.011, 0.126) | 0.0200 | -0.036 (-0.103, 0.031) | 0.3000 |
| Y7 N-acetylcarnosine (HILIC-neg) | 0.084  (0.035, 0.133) | 0.0007 | 0.082  (0.018, 0.146) | 0.0120 | -0.002  (-0.068, 0.064) | 0.9500 |
|  |  |  |  |  |  |  |
| **Panel C. Associations with total brain volume by race** | |  |  |  |  |  |
| **Metabolite** | **Black participants β**  **(95% CI)** | **P** | **White participants β (95% CI)** | **P** | **Interaction β (95% CI)** | **P interaction** |
| Y7 cotinine  (HILIC-pos) | -0.289  (-0.435, -0.142) | 0.0001 | -0.133 (-0.253, -0.012) | 0.0310 | 0.156 (-0.027, 0.338) | 0.0940 |
| Y7 biliverdin  (HILIC-pos) | -0.071 (-0.221, 0.08) | 0.3600 | 0.206 (0.078, 0.334) | 0.0017 | 0.276 (0.082, 0.471) | 0.0054 |
| Y7 C24:1 SM  (C8-pos) | -0.123  (-0.278, 0.033) | 0.1200 | -0.12 (-0.235, -0.004) | 0.0430 | 0.003 (-0.19, 0.197) | 0.9700 |
| Y7 C38:5 DAG  (C8-pos) | -0.188  (-0.352, -0.023) | 0.0260 | 0.06  (-0.063, 0.184) | 0.3400 | 0.248 (0.046, 0.449) | 0.0160 |
| Y7 C56:5 TAG  (C8-pos) | -0.145 (-0.297, 0.006) | 0.0600 | 0.001 (-0.122, 0.124) | 0.9900 | 0.146 (-0.047, 0.34) | 0.1400 |
| Y7 Ribothymidine  (C18-neg) | 0.247  (0.098, 0.396) | 0.0012 | 0.154  (0.036, 0.271) | 0.0110 | -0.093 (-0.281, 0.095) | 0.3300 |
| Y7 3-methylxanthine (HILIC-neg) | 0.118  (-0.046, 0.281) | 0.1600 | -0.03 (-0.156, 0.097) | 0.6500 | -0.147 (-0.352, 0.058) | 0.1600 |
| Y7 4-pyridoxate  (HILIC-neg) | -0.072  (-0.22, 0.076) | 0.3400 | 0.054 (-0.086, 0.194) | 0.4500 | 0.126 (-0.074, 0.327) | 0.2200 |
| erythronate/threonate (HILIC-neg) | 0.285  (0.126, 0.443) | 0.0005 | 0.052 (-0.069, 0.172) | 0.4000 | -0.233 (-0.431, -0.036) | 0.0210 |
| Y7 glycerate  (HILIC-neg) | 0.234  (0.086, 0.382) | 0.0020 | 0.043  (-0.076, 0.162) | 0.4800 | -0.191 (-0.38, -0.003) | 0.0470 |
| Y7 N-acetylcarnosine (HILIC-neg) | 0.045  (-0.131, 0.221) | 0.6100 | 0.155 (0.013, 0.296) | 0.0320 | 0.109 (-0.077, 0.296) | 0.2500 |
|  |  |  |  |  |  |  |
| **Panel D. Associations with cognitive performance by race** | |  |  |  |  |  |
| **Metabolite** | **Black participants β  (95% CI)** | **P** | **White participants β (95% CI)** | **P** | **Interaction β (95% CI)** | **P interaction** |
| Y7 cotinine  (HILIC-pos) | -0.159 (-0.21, -0.107) | 2.1E-09 | -0.067 (-0.111, -0.024) | 0.0025 | 0.091 (0.027, 0.156) | 0.0055 |
| Y7 biliverdin  (HILIC-pos) | 0.11  (0.064, 0.156) | 2.6E-06 | 0.048 (0.004, 0.092) | 0.0330 | -0.062 (-0.124, 0) | 0.0490 |
| Y7 C24:1 SM  (C8-pos) | -0.077  (-0.124, -0.03) | 0.0013 | -0.046 (-0.087, -0.005) | 0.0290 | 0.031 (-0.031, 0.093) | 0.3200 |
| Y7 C38:5 DAG  (C8-pos) | -0.12 (-0.172, -0.068) | 8.4E-06 | -0.031 (-0.071, 0.01) | 0.1400 | 0.089 (0.025, 0.154) | 0.0068 |
| Y7 C56:5 TAG  (C8-pos) | -0.088 (-0.136, -0.039) | 0.0004 | -0.047 (-0.088, -0.005) | 0.0270 | 0.041 (-0.022, 0.104) | 0.2000 |
| Y7 Ribothymidine  (C18-neg) | 0.057 (0.012, 0.102) | 0.0140 | 0.065 (0.022, 0.107) | 0.0029 | 0.008 (-0.053, 0.069) | 0.8000 |
| Y7 3-methylxanthine (HILIC-neg) | 0.117 (0.065, 0.169) | 1.0E-05 | 0.031 (-0.012, 0.074) | 0.1600 | -0.086 (-0.153, -0.019) | 0.0120 |
| Y7 4-pyridoxate  (HILIC-neg) | 0.062  (0.012, 0.111) | 0.0140 | 0.058 (0.011, 0.106) | 0.0150 | -0.003 (-0.07, 0.064) | 0.9300 |
| erythronate/threonate (HILIC-neg) | 0.06  (0.011, 0.108) | 0.0160 | 0.08 (0.038, 0.122) | 0.0002 | 0.02 (-0.044, 0.084) | 0.5400 |
| Y7 glycerate  (HILIC-neg) | 0.086  (0.039, 0.133) | 0.0003 | 0.094 (0.053, 0.135) | 8.4E-06 | 0.008 (-0.054, 0.069) | 0.8100 |
| Y7 N-acetylcarnosine (HILIC-neg) | 0.07  (0.016, 0.124) | 0.0110 | 0.094 (0.041, 0.146) | 0.0005 | 0.024 (-0.037, 0.084) | 0.4400 |

**Table S4.** Cognition associated metabolites (n=11) evaluated in relation to total brain volume and WMH. Linear regression adjusted for age, race, sex, recruitment site, and education.

| **Metabolite label** | **MRI_predictor** | **N** | **β** | **95% CI low** | **95% CI high** | **P** |
| --- | --- | --- | --- | --- | --- | --- |
| **Y7 Ribothymidine (C18-neg)** | **total brain** | 405 | **0.19** | **0.10** | **0.28** | **8.07E-05** |
| **Y7 cotinine (HILIC-pos)** | **total brain** | 404 | **-0.19** | **-0.29** | **-0.10** | **9.61E-05** |
| **Y7 C24:1 SM (C8-pos)** | **total brain** | 405 | **-0.12** | **-0.21** | **-0.03** | **1.12E-02** |
| **Y7 erythronate/threonate (HILIC-neg)** | **total brain** | 405 | **0.14** | **0.04** | **0.23** | **6.07E-03** |
| **Y7 glycerate (HILIC-neg)** | **total brain** | 405 | **0.12** | **0.02** | **0.21** | **1.42E-02** |
| Y7 N-acetylcarnosine (HILIC-neg) | total brain | 405 | 0.12 | -0.01 | 0.24 | 6.84E-02 |
| Y7 biliverdin (HILIC-pos) | total brain | 405 | 0.09 | -0.01 | 0.19 | 7.55E-02 |
| Y7 C56:5 TAG (C8-pos) | total brain | 405 | -0.06 | -0.15 | 0.04 | 2.47E-01 |
| Y7 3-methylxanthine (HILIC-neg) | total brain | 376 | 0.03 | -0.08 | 0.13 | 6.23E-01 |
| Y7 C38:5 DAG (C8-pos) | total brain | 405 | -0.03 | -0.13 | 0.07 | 5.95E-01 |
| Y7 4-pyridoxate (HILIC-neg) | total brain | 404 | -0.01 | -0.11 | 0.10 | 9.18E-01 |
| Y7 Ribothymidine (C18-neg) | WMH | 400 | -0.03 | -0.12 | 0.06 | 5.25E-01 |
| Y7 cotinine (HILIC-pos) | WMH | 399 | 0.06 | -0.03 | 0.16 | 1.73E-01 |
| Y7 C24:1 SM (C8-pos) | WMH | 400 | 0.00 | -0.08 | 0.09 | 9.20E-01 |
| Y7 erythronate/threonate (HILIC-neg) | WMH | 400 | -0.05 | -0.14 | 0.04 | 2.84E-01 |
| Y7 glycerate (HILIC-neg) | WMH | 400 | -0.07 | -0.16 | 0.02 | 1.34E-01 |
| Y7 N-acetylcarnosine (HILIC-neg) | WMH | 400 | -0.04 | -0.16 | 0.08 | 4.95E-01 |
| Y7 biliverdin (HILIC-pos) | WMH | 400 | -0.06 | -0.16 | 0.03 | 1.82E-01 |
| Y7 C56:5 TAG (C8-pos) | WMH | 400 | 0.01 | -0.09 | 0.11 | 7.62E-01 |
| Y7 3-methylxanthine (HILIC-neg) | WMH | 372 | 0.01 | -0.09 | 0.10 | 8.85E-01 |
| Y7 C38:5 DAG (C8-pos) | WMH | 400 | -0.02 | -0.11 | 0.08 | 7.19E-01 |
| Y7 4-pyridoxate (HILIC-neg) | WMH | 399 | -0.07 | -0.17 | 0.02 | 1.36E-01 |

**Table S5**. Associations of cognition- and total brain volume-associated metabolites with anatomically defined regional brain volumes. Linear regression models examined associations of the five metabolites associated with both Year 30 cognitive performance and total brain volume with parietal, frontal, temporal, occipital, and limbic gray matter volumes and hippocampal volume. Models were adjusted for age, race, sex, recruitment site, and education. All regional volumes were normalized to intracranial volume and standardized for analysis. A Bonferroni-corrected significance threshold of P < 0.00167 was applied to account for 30 regional comparisons (5 metabolites × 6 brain regions).

| **Brain region** | **Metabolite** | **β** | | **95% CI** | | **P value** |
| --- | --- | --- | --- | --- | --- | --- |
| Parietal | cotinine | -0.122 | -0.217 | | -0.026 | 1.24E-02 |
|  | C24:1 SM | -0.036 | -0.129 | | 0.056 | 4.41E-01 |
|  | Ribothymidine | 0.068 | -0.026 | | 0.161 | 1.57E-01 |
|  | erythronate/threonate | 0.130 | 0.033 | | 0.227 | 8.72E-03 |
|  | glycerate | 0.112 | 0.019 | | 0.206 | 1.89E-02 |
| Frontal | cotinine | -0.151 | -0.247 | | -0.055 | 2.17E-03 |
|  | C24:1 SM | -0.080 | -0.174 | | 0.013 | 9.17E-02 |
|  | **Ribothymidine** | **0.161** | **0.067** | | **0.254** | **8.16E-04** |
|  | erythronate/threonate | 0.060 | -0.039 | | 0.158 | 2.35E-01 |
|  | glycerate | 0.080 | -0.015 | | 0.175 | 9.88E-02 |
| Temporal | cotinine | -0.109 | -0.211 | | -0.007 | 3.72E-02 |
|  | C24:1 SM | -0.048 | -0.147 | | 0.051 | 3.45E-01 |
|  | Ribothymidine | 0.079 | -0.021 | | 0.179 | 1.20E-01 |
|  | erythronate/threonate | 0.063 | -0.042 | | 0.167 | 2.39E-01 |
|  | glycerate | 0.074 | -0.026 | | 0.175 | 1.47E-01 |
| Occipital | cotinine | -0.117 | -0.219 | | -0.014 | 2.57E-02 |
|  | C24:1 SM | -0.045 | -0.144 | | 0.054 | 3.75E-01 |
|  | Ribothymidine | 0.080 | -0.020 | | 0.181 | 1.18E-01 |
|  | erythronate/threonate | 0.128 | 0.024 | | 0.232 | 1.60E-02 |
|  | glycerate | 0.136 | 0.036 | | 0.236 | 8.03E-03 |
| Limbic | cotinine | -0.126 | -0.223 | | -0.030 | 1.05E-02 |
|  | C24:1 SM | -0.046 | -0.140 | | 0.047 | 3.32E-01 |
|  | Ribothymidine | 0.138 | 0.044 | | 0.232 | 4.21E-03 |
|  | erythronate/threonate | 0.057 | -0.042 | | 0.157 | 2.55E-01 |
|  | glycerate | 0.079 | -0.017 | | 0.174 | 1.06E-01 |
| Hippocampal | cotinine | -0.148 | -0.247 | | -0.049 | 3.57E-03 |
|  | C24:1 SM | -0.018 | -0.114 | | 0.079 | 7.16E-01 |
|  | Ribothymidine | 0.130 | 0.033 | | 0.227 | 8.57E-03 |
|  | erythronate/threonate | 0.046 | -0.055 | | 0.148 | 3.70E-01 |
|  | glycerate | 0.034 | -0.064 | | 0.132 | 5.00E-01 |

**Table S6**. **Primary and smoking-adjusted mediation results for associations between plasma metabolites, total brain volume, and cognitive performance.** Indirect, direct, and total effects presented with 95% confidence intervals (CIs) estimated using `medeff` in Stata with 10,000 quasi-Bayesian simulations. Effects represent differences in standardized Year 30 cognitive performance per 1-SD higher Year 7 metabolite level. The mediator was the standardized, total brain volume/intracranial volume ratio. Primary models adjusted for age, race, sex, field center, and education. Smoking-adjusted models additionally included categorical Year 7 smoking status in both the mediator and outcome equations. Mediator and outcome regressions included 405 and 398 participants, respectively, except for cotinine (404 and 397), in both model sets. Indirect-effect attenuation was calculated from unrounded estimates as 100 × (1-(smoking-adjusted indirect effect / primary indirect effect)). Abbreviations: CI, confidence interval; SM, sphingomyelin.

| **Metabolite** | **Model** | **Indirect effect (95% CI)** | **Direct effect (95% CI)** | **Total effect (95% CI)** | **% Mediated** | **% Attenuation** |
| --- | --- | --- | --- | --- | --- | --- |
| Ribo-thymidine | Primary | 0.025  (0.007, 0.048) | 0.122  (0.037, 0.208) | 0.147  (0.061, 0.233) | 17 |  |
|  | Smoking-adjusted | 0.021  (0.004, 0.043) | 0.120  (0.028, 0.212) | 0.140  (0.047, 0.232) | 15 | 18 |
| Cotinine | Primary | -0.029  (-0.055, -0.009) | -0.055  (-0.145, 0.035) | -0.084  (-0.172, 0.005) | 33 |  |
|  | Smoking-adjusted | -0.024  (-0.052, -0.004) | -0.032  (-0.147, 0.084) | -0.056  (-0.172, 0.060) | 29 | 15 |
| C24:1 SM | Primary | -0.018  (-0.039, -0.003) | -0.058  (-0.142, 0.027) | -0.076  (-0.160, 0.010) | 22 |  |
|  | Smoking-adjusted | -0.015  (-0.034, -0.001) | -0.054  (-0.138, 0.031) | -0.069  (-0.154, 0.016) | 20 | 16 |
| Erythronate/ Threonate | Primary | 0.017  (0.003, 0.037) | 0.193  (0.107, 0.279) | 0.210  (0.122, 0.297) | 8 |  |
|  | Smoking-adjusted | 0.014  (0.0001, 0.032) | 0.190  (0.103, 0.277) | 0.203  (0.115, 0.291) | 7 | 21 |
| Glycerate | Primary | 0.017  (0.002, 0.036) | 0.136  (0.053, 0.220) | 0.153  (0.068, 0.238) | 11 |  |
|  | Smoking-adjusted | 0.014  (0.001, 0.032) | 0.132  (0.049, 0.217) | 0.146  (0.061, 0.230) | 9 | 17 |
